# Accuracy of Diagnosis of Benign Vocal Fold Lesions among Ear, Nose, and Throat Residents

**DOI:** 10.1055/s-0044-1787168

**Published:** 2024-07-05

**Authors:** Mohamed Mesfer Alessa, Sultan Bin Obaid, Faisal Aldayel, Rakan Almetary, Khaled Alhussinan, Hassan Assiri, Mohamed Farahat

**Affiliations:** 1 Department of Otolaryngology, Faculty of Medicine, King Saud University, Riyadh, Saudi Arabia; 2 Department of Otolaryngology, Head and Neck Surgery, King Abdulaziz University Hospital, King Saud University, Riyadh, Saudi Arabia; 3 Department of Otolaryngology, Research Group of Voice, Swallowing and Communication Disorders, Faculty of Medicine, King Saud University, Riyadh, Saudi Arabia

**Keywords:** medical residency, vocal folds, training

## Abstract

**Introduction** Benign vocal fold lesions (BVFLs) disrupt the superficial lamina propria, impacting vocal fold pliability. Investigating the diagnostic competency of ear, nose, and throat (ENT) residents in identifying BVFLs, we hypothesize that factors such as residency year, subspecialty focus, and training center influence diagnostic accuracy.

**Objectives** To assess the accuracy of diagnosis of BVFLs among ENT residents and to correlate diagnostic accuracy with the year of residency.

**Methods** An observational cross-sectional study included all ENT residents in Saudi Arabia. It was performed in August and September 2021 using a self-administered online questionnaire that assessed demographic variables and included three images with open-ended questions on diagnosing vocal fold lesions.

**Results** A total of 61 ENT residents (62.3% male) were included in this study. The images of vocal fold cyst, vocal fold polyp, and vocal fold nodule were correctly diagnosed by 60.7%, 88.5%, and 91.8% of residents, respectively. There was a correlation between the year of residency and the accuracy of diagnosing a vocal fold cyst ( *p* = 0.029). Residents interested in laryngology correctly diagnosed all three lesions more frequently than other residents.

**Conclusion** The ability of residents to diagnose vocal fold cysts was moderate. In particular, the senior residents were able to diagnose polyps and nodules with excellent accuracy.

## Introduction

Vocal folds are composed of five layers; each layer has its own anatomical and mechanical characteristics. From superficial to deep, the layers consist of very thin squamous epithelium, lamina propria that is divided into three layers based on the density of elastic and collagenous fibers (superficial [Reinke space], intermediate, and deep), and thyroarytenoid muscles (the bulk). Those five layers can be mechanically classified into three parts: the “cover,” consisting of the epithelium and lamina propria superficial layers; the “transition,” composed of intermediate and deep layers of the lamina propria; and the “body,” consisting of the vocalis muscle. 1 2

Benign vocal fold lesions (BVFLs) are non-cancerous growth of aberrant tissue on the folds of the vocal folds, including Singer nodules, polyps, cysts, and others. It develops in the superficial lamina propria, the vibratory layer of the vocal fold, where the fibrous components are loose and can be likened to a mass of soft gelatin. If BVFLs occur, they may affect pliability and mucosal waves during phonation and cause glottic insufficiency. 3 4 The underlying mechanism of BVFLs development is unclear, whether it is caused primarily by phonotrauma or by an unsolicited injury that leads to overcompensation and secondary lesions. Inflammation that is related to vocal misuse, laryngopharyngeal reflux, and phonotrauma also plays an essential role in the development of BVFLs. 3 5 6 7 Various symptoms are associated with BVFLs, including hoarseness, voice fatigue, effortful speech, and voice strain. Rarely, patients with large lesions may also experience airway obstruction. 3 In 2018, the clinical practice guidelines stated that any patient presenting with voice changes for 4 weeks should undergo diagnostic laryngoscopy performed by a clinician, before starting any treatment. 7 Videostroboscopy and direct fiberoptic visualization are the gold-standard methods for diagnosing the presence of BVFLs. 3 It has been found that the use of narrow band imaging (NBI)/white light improves the detection rate of vocal fold cysts, while NBI is not found to significantly aid in the detection of polyps. 8 Despite advancements in technology and the use of both white halogen and stroboscopic light, inconsistencies were observed between the preoperative diagnosis and the intraoperative diagnosis of BVFLs in 36% of patients. 9

The management of BVFLs varies from behavioral intervention (voice therapy, good vocal fold hygiene practice, and treatment of exacerbating factors like laryngopharyngeal reflux), steroid injection to the lesion, or surgery using a micro flap or laser. In some cases, however, conservative management is less likely to produce positive results, such as in the case of polyps. 10 There is no consensus regarding the use of voice therapy for lesions other than nodules, anti-reflux medications, and intravenous steroids. 3 11

For optimal and patient-specific treatment, the clinician should be familiar with the anatomy, physiology, and functional aspects of those lesions. 12 Determining the competency of ear, nose, and throat (ENT) residents in diagnosing BVFLs will be useful, as they are responsible for the accurate diagnosis that helps guide appropriate therapy. Thus, correlating their capability with each training program may help assess the competency of residency programs. Therefore, this study assessed the capability of ENT residents in diagnosing BVFLs since they diagnose and treat these conditions. We hypothesized that the year of residency, subspeciality of interest, and residency center would affect the residents' accuracy of diagnosis.

## Methods

This was a quantitative cross-sectional study approved by the Institutional Review Board of the College of Medicine of King Saud University (No. E-21-6071). The informed consent form stated that participation was voluntary, data were collected for research purposes only, self-identifying information (i.e., name, university number, and phone number) would not be collected, and confidentiality and privacy would be maintained during all study phases. Participants were included only if they provided informed consent.

The study was conducted by contacting the chief resident in each ENT center of Saudi Arabia (Central, Eastern, Western, and Southern regions) using an online questionnaire. The questionnaire consisted of two sections: one on demographic data and another that had questions assessing the diagnostic capability of ENT residents. This study included ENT residents practicing in Saudi Arabia, excluding R1 (postgraduate year 1). Our study period went from August 1 ^st^ , 2021, to September 30 ^th^ , 2021.

**Fig. 1:**
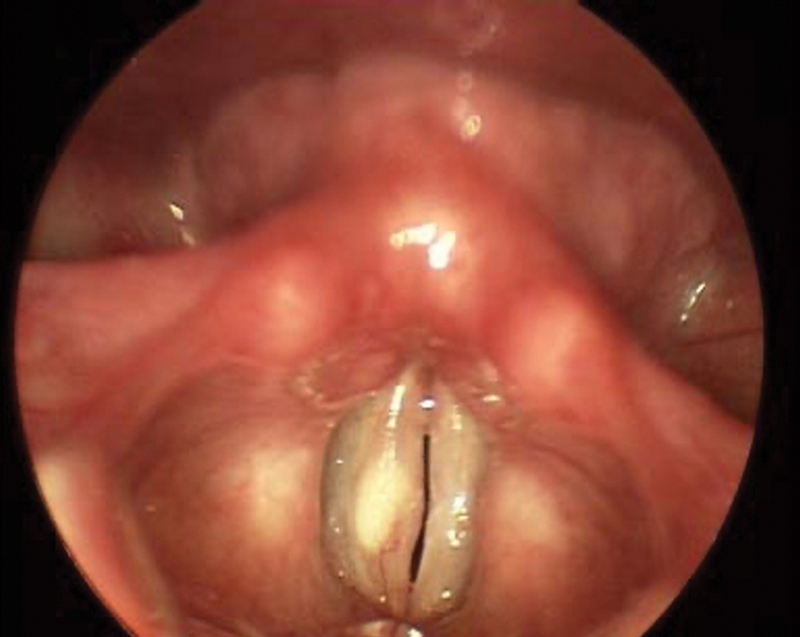
Vocal fold cyst.

The residents were provided with three images ( Figs. 1 2 3 ) and using an open-ended format; They were asked to identify the lesion that was most likely a benign vocal fold lesion. This questionnaire was first sent to the residents on August 2 ^nd^ , a reminder was sent on August 9 ^th^ , and another reminder was sent on August 16 ^th^ . The images that were provided were piloted first among 10 expert ENT consultants for verification of the correct diagnosis.

**Fig. 2:**
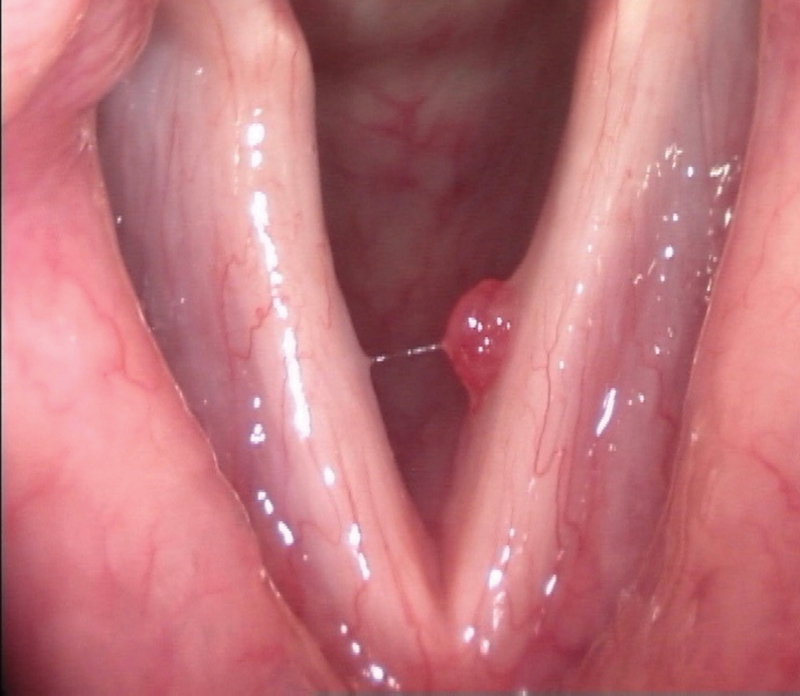
Vocal fold polyp.

**Fig. 3:**
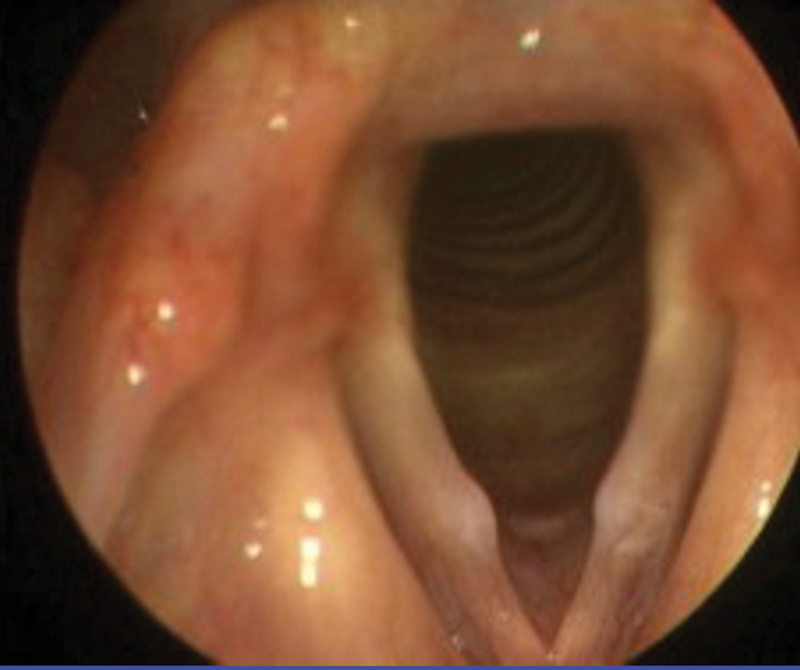
Vocal folds nodules.

All data collected in this study were analyzed using the IBM SPSS Statistics for Windows, version 26.0 (IBM Corp., Armonk, NY, USA) software. Descriptive statistics (means, standard deviations, frequencies, and percentages) were used to describe the quantitative and categorical variables. A bivariate statistical analysis was conducted, using appropriate independent t-tests and one-way analysis of variance statistical tests according to the type of study and outcome variables. A *p* -value < 0.05 and a 95% confidence interval were used to report the statistical significance and the precision of the results, respectively.

## Results

Overall, 188 eligible residents received the survey and 61 completed it, with a response rate of 32.4%. The percentage of male and female respondents was 62.3% and 37.7%, respectively. Regarding the region, 52.5%, 19.7%, 13.1%, and 14.8% of residents were from the Central, Western, Southern, and the Eastern regions, respectively. Regarding the year of residency, 16.4%, 26.2%, 31.1%, and 26.2% were 2nd-, 3rd-, 4th-, and 5th-year residents, respectively. The proportions of residents interested in facial plastic surgery, head and neck surgery, pediatric ENT, otology, rhinology, and laryngology were 21.3%, 29.5%, 14.8%, 14.8%, 13.1%, and 6.6%, respectively ( Table 1 ).

**Table 1:** Demographic characteristics of respondents (n = 61)

Variable		n (%)	SD
Sex	Male	38 (62.3)	0.489
	Female	23 (37.7)	
Region	Central	32 (52.5)	1.121
	Western	12 (19.7)	
	Southern	8 (13.1)	
	Eastern	9 (14.8)	
Year of residency	Year 2	10 (16.4)	1.044
	Year 3	16 (26.2)	
	Year 4	19 (31.1)	
	Year 5	16 (26.2)	
Subspeciality of interest	Facial plastic surgery	13 (21.3)	1.561
	Head and neck surgery	18 (29.5)	
	Pediatric ear, nose, and throat	9 (14.8)	
	Otology	9 (14.8)	
	Rhinology	8 (13.1)	
	Laryngology	4 (6.6)	

**Abbreviation:** SD, standard deviation.

For the first image ( Fig. 1 ), which was a vocal fold cyst, 60.7% made the correct diagnosis, while 9.8% made an incorrect diagnosis of vocal fold edema. Regarding the second image ( Fig. 2 ), 88.5% made the correct diagnosis of a vocal fold polyp, while 11.5% made incorrect diagnoses. For the third image ( Fig. 3 ), 91.8% made the correct diagnosis of a vocal fold nodule ( Table 2 ).

**Table 2:** Respondents' answers and means (n = 61)

Parameters		n	%
First image	Reinke edema	3	4.9
	Vocal fold cyst	37	60.7
	Right vocal fold lesion	3	4.9
	Edema	6	9.8
	Right vocal fold nodule	2	3.3
	Paralysis	1	1.6
	Leucoplakia	3	4.9
	Right vocal fold mass	1	1.6
	Granuloma	1	1.6
	Sulcus	2	3.3
	Laryngeal keratosis	2	3.3
Second image	Nodule	2	3.3
	Vocal fold polyp	54	88.5
	Polyp/granuloma	1	1.6
	Vocal fold papilloma	3	4.9
	Fleshy vocal mass	1	1.6
Third image	Vocal fold nodules	56	91.8
	Bilateral vocal fold cyst	2	3.3
	Polyp	2	3.3
	Contact granuloma	1	1.6

The year of residency correlated with the accuracy of diagnosis of the vocal fold cyst ( *p* = 0.029). Although this was the only statistically significant finding, some other findings should be highlighted. When evaluating the vocal fold cyst, 55.5% and 41.6% of the residents from the Eastern and Western regions, respectively, made incorrect diagnoses. Moreover, 62.5% and 44% of those with an interest in rhinology and head and neck surgery, respectively, made incorrect diagnoses when evaluating the vocal fold cyst. Those with an interest in laryngology showed higher percentages of correct diagnoses for the three lesions compared with other residents who had other subspecialty interests ( Table 3 ).

**Table 3:** Correlation of correct answers with demographics:

		First image			Second image			Third image		
Variable		VFC	IA	*p*	VFP	IA	*P*	VFN	IA	*p*
Sex	Male	23	15	0.247	33	5	0.845	33	5	0.572
	Female	14	9		21	2		22	1	
Region	Central	20	6	0.34	26	6	0.946	29	3	0.666
	Western	7	5		11	1		11	1	
	Southern	6	2		8	0		8	0	
	Eastern	4	5		9	0		8	1	
Year of residency	Year 2	3	7	0.029	6	4	0.192	8	2	0.122
	Year 3	9	7		14	2		15	1	
	Year 4	12	7		19	0		19	0	
	Year 5	13	3		15	0		14	2	
Subspeciality of interest	Facial plastic surgery	10	3	0.427	10	3	0.649	10	3	0.477
	Head and neck surgery	10	8		17	1		17	1	
	Pediatric ear nose throat	6	3		9	0		9	0	
	Otology	5	4		8	1		8	1	
	Rhinology	3	5		6	2		8	0	
	Laryngology	3	1		4	0		4	0	

**Abbreviations:** IA, incorrect answer; VFC, vocal fold cyst; VFN, vocal fold nodule; VFP, vocal fold polyp.

All three images were correctly diagnosed by 52.5% of the sample. Regarding the regions, those from the Southern region presented the highest percentage (75%) of correct diagnoses for all 3 images. Although the results were not significant, 20% of the 2nd-year residents and 62.5% of the 5th-year residents made correct diagnoses for all 3 images. Of those with an interest in rhinology and otology, 37.5% and 44.4% made correct diagnoses, respectively, while 75% of those with an interest in laryngology made correct diagnoses of all 3 images ( Table 4 ).

**Table 4:** Mean number and correlation of residents who answered all questions correctly

Variable		Correct	≥ 1 wrong answer	*p* -value
Region	Central	15	17	0.488
	Western	7	5	
	Southern	6	2	
	Eastern	4	5	
Year of residency	Year 2	2	8	0.123
	Year 3	8	8	
	Year 4	12	7	
	Year 5	10	6	
Subspeciality of interest	Facial plastic surgery	6	7	0.427
	Head and neck surgery	10	8	
	Pediatric ear nose throat	6	3	
	Otology	4	5	
	Rhinology	3	5	
	Laryngology	3	1	
	Total (%)	32 (52.5)	29 (47.5)	

## Discussion

In this study, we aimed to assess the Saudi Arabian ENT residents' ability to diagnose BVFLs accurately. Further, we aimed to correlate the findings with the residency level. Our findings may reflect the abilities of the residents to diagnose vocal fold lesions without clinical context, indicating how frequently these conditions are observed in clinics or surgical theatres. Subsequently, they may reveal the shortcomings to residency supervisors, thus enabling the implementation of resources and utilities to improve the outcomes of otolaryngology residency training programs in Saudi Arabia. To the best of our knowledge, this was the first study to assess the accuracy of diagnoses of vocal fold lesions among ENT residents in Saudi Arabia.

We found that a vocal fold cyst was the type that was diagnosed least accurately, while vocal fold polyps and nodules were diagnosed correctly by 88.5% and 91.8% of the residents, respectively. While reviewing the literature, we found that several vocal fold diseases are difficult to diagnose even using stroboscopic light. Sulcus vocalis, submucosal cysts, pseudocysts, and mucosal bridges are some examples. 13 Furthermore, one of the difficulties in diagnosing a vocal fold cyst is that, unlike other benign vocal fold lesions, cysts are usually lined by normal respiratory epithelium, which rarely becomes ulcerated. 14 Additionally, out of all the cases of epidermoid cysts, only 10% were diagnosed on initial examination, and 55% of the cases were only suspected because of the presence of localized subtle fullness on a point corresponding to the middle third of the membranous portion of a vocal fold. 15 This may explain the lower percentage of accurate diagnoses among ENT residents for a vocal fold cyst. Cipriani et al. studied the clinical and pathological spectrum of BVFLs and reviewed the reliability of histological diagnoses in these cases, concluding that “a polyp, nodule, or Reinke edema is neither clinically reproducible nor histologically unique.” They also stated that since histological features can overlap, shared stroboscopic features may lead to different interpretations of cysts and nodules. 14 Although not statistically significant, residents interested in laryngology showed higher percentages of correct diagnoses compared to others, and that might be explained by further reading and educational exposure. More research is required in the area to explore potential positive explanations which may influence our programs.

We found that a resident's competency to diagnose BVFLs accurately correlated significantly with the year of residency. Further, a higher percentage of senior residents (4th and 5th years) correctly diagnosed the other 2 conditions. A study conducted to assess the progression of reliability and competency in the use of trans nasal laryngoscope among the ENT residents showed significant improvements in the diagnoses of vocal fold immobility, subglottic stenosis, laryngeal mass, vocal fold abnormalities using intraclass correlation in residents, according to the residency year. 16

The results of another study that was conducted to develop an objective technical skills assessment tool for residents' surgical performance in pediatric laryngoscopy and rigid bronchoscopy were consistent with these findings. 17

Our findings highlight that the residents diagnosed vocal fold nodules and polyps more easily than vocal fold cysts, indicating the prevalence of these two conditions in their training programs. However, no study in the literature has assessed the exposure of residents to vocal fold lesions. Thus, we considered that epidemiologically, the two conditions were more common than vocal fold cysts.

Poels et al. studied the consistency in the clinical diagnoses of BVFLs identified at preoperative and intraoperative examinations. Interestingly, in their paper, vocal fold nodules and polyps were prevalent in more than half of their study sample, while vocal folds cysts showed one of the lowest prevalence, alongside sulci vocalis and vergetures. 9 Another research assessed the age, sex distribution, symptomatology, areas of involvement, and prognosis of the most prevalent forms of benign laryngeal lesions. In their study sample, 40.47% presented vocal fold polyps and 28.57% presented vocal fold nodules, while a vocal fold cyst was not encountered. 18

We also noted that, although not statistically significant, higher percentages of misdiagnosed vocal fold cyst cases were noted in the Eastern and Western regions. Nonetheless, this higher percentage may point out some difficulties that ENT residents in these regions may face, such as the lower number of subspecialized staff in regions where they can accept a higher number of such cases.

There were some limitations to our study. In the questionnaire, we assessed the accuracy of diagnoses using images instead of videos. Although the pictures were clear and of high quality, videos would have been better to visualize and assess the anomalies accurately. Despite diligent efforts to gather responses, the study encountered limitations with a moderate response rate of 32.4%, involving 61 out of 188 surveyed residents. Additionally, the absence of participation from the northern region further constrained the geographic diversity of the sample. These limitations potentially restrict the generalizability of findings to the entire resident population of Saudi Arabia. Future studies with broader participation across regions are recommended for a more comprehensive understanding of the measured aspects.

## Conclusion

The abilities of the residents to diagnose vocal fold cysts were moderate. This may have been because of the low prevalence of this condition compared with the other two. However, they showed excellent capabilities regarding the diagnosis of polyps and nodules, especially at the senior residency level. Regions and subspecialties were not statistically indicative of each resident's ability to accurately diagnose those conditions.

It is recommended that future researchers investigate the reasons that yield higher percentages of incorrect diagnoses among ENT residents and use qualitative methods to gain better insights into a resident's opinions and thoughts. Ear, nose, and throat training centers should offer conferences and lectures regarding vocal fold lesions and expose the residents to more cases in clinics.

## References

[1] Hirano M Morphological structure of the vocal cord as a vibrator and its variations Folia Phoniatr (Basel) 1974 26 02 89 94 10.1159/000263771 4845615

[2] Hirano M Kakita Y Ohmaru K Kurita S Structure and mechanical properties of the vocal fold Speech and Language. 1982 7 271 297. Doi: 10.1016/B978-0-12-608607-2.50015-7

[3] Naunheim M R Carroll T L Benign vocal fold lesions: update on nomenclature, cause, diagnosis, and treatment Curr Opin Otolaryngol Head Neck Surg 2017 25 06 453 458. Doi: 10.1097/MOO.0000000000000408 29099730 10.1097/MOO.0000000000000408

[4] Malik P Yadav S PS Sen R The clinicopathological study of benign lesions of vocal cords Indian J Otolaryngol Head Neck Surg 2019 71 01 212 220 10.1007/s12070-017-1240-0 PMC6848543 31741962

[5] Wang L Tan J J Wu T Association between laryngeal pepsin levels and the presence of vocal fold polyps Otolaryngol Head Neck Surg 2017 156 01 144 151 10.1177/019459981667671 28045635

[6] Lechien J R Saussez S Nacci A Association between laryngopharyngeal reflux and benign vocal folds lesions: A systematic review Laryngoscope 2019 129 09 E329 E341. Doi: 10.1002/lary.27932 30892725 10.1002/lary.27932

[7] Kunduk M McWhorter A J True vocal fold nodules: the role of differential diagnosis Curr Opin Otolaryngol Head Neck Surg 2009 17 06 449 452. Doi: 10.1097/MOO.0b013e3283328b6d 19779347 10.1097/MOO.0b013e3283328b6d

[8] Dippold S Nusseck M Richter B Echternach M The use of narrow band imaging for the detection of benign lesions of the larynx Eur Arch Otorhinolaryngol 2017 274 02 919 923 10.1007/s00405-016-4300-2 27631509

[9] Poels P J de Jong F I Schutte H K Consistency of the preoperative and intraoperative diagnosis of benign vocal fold lesions J Voice 2003 17 03 425 433. Doi: 10.1067/S0892-1997(03)00010-9 14513965 10.1067/s0892-1997(03)00010-9

[10] Lee Y S Lee D H Jeong G-E Treatment efficacy of voice therapy for vocal fold polyps and factors predictive of its efficacy J Voice 2017 31 01 1.2E11 1.2E15. Doi: 10.1016/j.jvoice.2016.02.014 27017066 10.1016/j.jvoice.2016.02.014

[11] Sulica L Behrman A Management of benign vocal fold lesions: a survey of current opinion and practice Ann Otol Rhinol Laryngol 2003 112 10 827 833 10.1177/00034894031120100 14587971

[12] Bohlender J Diagnostic and therapeutic pitfalls in benign vocal fold diseases GMS Curr Top Otorhinolaryngol Head Neck Surg 2013 12 Doc01. Doi: 10.3205/cto000093 24403969 10.3205/cto000093 PMC3884536

[13] Hernando M Cobeta I Lara A García F Gamboa F J Vocal pathologies of difficult diagnosis J Voice 2008 22 05 607 610. Doi: 10.1016/j.jvoice.2006.12.011 17324554 10.1016/j.jvoice.2006.12.011

[14] Cipriani N A Martin D E Corey J P The clinicopathologic spectrum of benign mass lesions of the vocal fold due to vocal abuse Int J Surg Pathol 2011 19 05 583 587 10.1177/1066896911411480 21685134

[15] Dikkers F G Nikkels P G Benign lesions of the vocal folds. Clinical and histopathological aspects Ann Otol Rhinol Laryngol 1995 104 698 703 10.1177/000348949510400905 7661518

[16] Brook C D Platt M P Russell K Grillone G A Aliphas A Noordzij J P Time to competency, reliability of flexible transnasal laryngoscopy by training level: a pilot study Otolaryngol Head Neck Surg 2015 152 05 843 850 10.1177/0194599815572792 25788339

[17] Ishman S L Brown D J Boss E F Development and pilot testing of an operative competency assessment tool for pediatric direct laryngoscopy and rigid bronchoscopy Laryngoscope 2010 120 11 2294 2300. Doi: 10.1002/lary.21067 20939072 10.1002/lary.21067

[18] Hegde M C Kamath M P Bhojwani K Peter R Babu P R Benign lesions of larynx-A clinical study Indian J Otolaryngol Head Neck Surg 2005 57 01 35 38. Doi: 10.100/BF02907624 23120121 10.1007/BF02907624 PMC3451554

